# First Molecular Detection of Orthohantaviruses (*Orthohantavirus hantanense* and *O. jejuense*) in Trombiculid Mites from Wild Rodents in the Republic of Korea

**DOI:** 10.3390/pathogens14121260

**Published:** 2025-12-09

**Authors:** Seong Yoon Kim, Hak Seon Lee, Hyunyoung Yoon, Hee Il Lee

**Affiliations:** 1 Division of Vectors and Parasitic Diseases, Bureau of Infectious Disease Diagnosis Control, Korea Disease Control and Prevention Agency, 187 Osongsaengmyeong 2-ro, Cheongwon-gun, Cheongju-si 363-951, Republic of Korea; gunbo0402@korea.kr (S.Y.K.); hslee8510@korea.kr (H.S.L.); yhypia@korea.kr (H.Y.); 2 Department of Veterinary Medicine & Public Health, Armed Forces Medical Research Institute, 90bun, Jaun-ro, Yuseong-gu, Daejeon 34059, Republic of Korea

**Keywords:** Orthohantavirus, trombiculid mites, *Orthohantavirus hantanense*, *O. jejuense*, potential vector

## Abstract

Orthohantaviruses are zoonotic pathogens that cause severe diseases in humans primarily through inhalation of aerosols from rodent excreta. Recent studies suggest that ectoparasites may be potential vectors for Orthohantaviruses. This study aimed to obtain molecular evidence of Orthohantavirus in trombiculid mites collected from wild rodents. In April 2025, 4963 trombiculid mites were collected from 128 wild rodents captured in 17 regions of the Republic of Korea (ROK). Among them, 1660 mites were grouped into 204 pools by collection sites and tested for the Orthohantavirus RNA using quantitative real-time polymerase chain reaction (PCR) and reverse transcription-nested PCR. OrthoHantavirus RNA was detected in seven trombiculid mite pools, with a minimum infection rate of 0.4/100 mites. *Orthohantavirus hantanense* was identified in trombiculid mites from Cheongju, Gimcheon, and Yeongdeok, while *O. jejuense* was identified in trombiculid mites from Boryeong and Jeongeup. Notably, Orthohantaviruses were not detected in the host rodents of the Orthohantavirus-positive trombiculid mites from Cheongju, Jeongeup, and Gimcheon, indicating host-independent infection. This study is the first to report detection of Orthohantaviruses, *O. hantanense* and *O. jejuense*, from trombiculid mites in the ROK. The host-independent infection suggests that these mites could serve as independent vectors/reservoirs for Orthohantaviruses, distinct from previously known transmission routes.

## 1. Introduction

Orthohantaviruses are zoonotic pathogens belonging to the genus *Orthohantavirus* within the family *Hantaviridae*. Currently, 38 Orthohantavirus species have been reported, including *O. hantanense*, *O. seoulense*, *O. puumalaense*, *O. dobravaense*, and *O. andesense* [1]. Among these, at least 24 species are known to cause severe and often fatal diseases in humans [2], including hemorrhagic fever with renal syndrome (HFRS) with case fatality rates ranging from 1% to 15% in Eurasia, and the more severe hantavirus pulmonary syndrome (HPS) with case fatality rates between 35% and 50% in the Americas [3]. Several small mammals, including rodents, shrews, moles, and bats, are primary reservoirs of hantavirid species [4], and the host species vary by region [5]. HFRS and HPS are transmitted to humans through inhalation of infectious particles in the form of aerosols from Orthohantavirus-contaminated feces, urine, or saliva, or through bites from infected hosts [4].

In the Republic of Korea (ROK), an average of 402 cases of HFRS with a fatality rate of 0.8% were reported annually from 2011 to 2024, with more than 60% of cases occurring between October and December [6]. HFRS caused by *O. hantanense* and *O. seoulense* [7] is a major rodent-borne disease along with scrub typhus and leptospirosis in the ROK [8]. *Orthohantavirus hantanense* was first isolated from the lung of a wild rodent, *Apodemus agrarius*, collected in 1976 in Gyeonggi Province, ROK [9]. Since then, various molecular and serological studies have identified the Orthohantaviruses in small mammals, including *A. peninsulae* [7], *Rattus norvegicus* [10], and *Craseomys regulus* [11]; shrews, *Crocidura lasiura* [12] and *C. shantungensis* [13]; and bats, *Rhinolophus ferrumequinum* [14].

Trombiculid mites (family Trombiculidae), belonging to the class Arachnida, are arthropods that serve as the primary vectors of scrub typhus in the ROK. 63 species of trombiculid mites have been reported in the ROK, with *Leptotrombidium pallidum*, *L. scutellare*, and *L. palpale* being the dominant species [15]. These trombiculid mites inhabit the soil and undergo a life cycle consisting of egg, larva, nymph, and adult stages. They are parasitic only during the six-legged larval stage, feeding on the tissue fluid and lymph of hosts, such as rodents or birds, for approximately 2 to 10 days. Transmission of scrub typhus can occur when a pathogen-infected larva bites a human, who serves as an accidental host. In contrast, the eight-legged nymphs and adults are free-living predators in the soil, feeding on other arthropods or eggs; however, relatively little is known about their taxonomy and ecology during these stages [16]. Recently, several studies have reported the potential role of ectoparasites as vectors of Orthohantaviruses. Jiang et al. [17] compared the L segments of Orthohantaviruses from patients with HFRS, wild rodents, and ectoparasites in Qingdao, China. They found that Orthohantaviruses from gamasid and trombiculid mites were identical to those from their hosts, suggesting that mite-mediated transmission may facilitate HFRS infection in humans. Furthermore, Houck, Qin & Roberts [18] detected Orthohantaviruses in ticks and trombiculid mites parasitizing on wild rodents collected in Texas, USA, and also found the Orthohantavirus in a free-living trombiculid mite collected from the soil at the same collection site. These findings suggest that trombiculid mites could be involved in the transmission cycle between rodents and humans. To better understand the potential vectors of Orthohantavirus, we conducted nationwide molecular surveillance of Orthohantaviruses in trombiculid mites collected from wild rodents.

## 2. Materials and Methods

### 2.1. Ethics Statement

All animal handling and experimental procedures were approved by the Institutional Biosafety Committee (approval number: KDCA-IBC-2024-034) and the Institutional Animal Care and Use Committee (approval number: KDCA-IACUC-24-019) of the Korea Disease Control and Prevention Agency (KDCA). After obtaining permission under the Wildlife Protection and Management Act, all wild rodents were captured and euthanized in accordance with the approved protocols. The species collected in this study are not listed as endangered or protected in the ROK.

### 2.2. Rodent Trapping and Trombiculid Mite Sampling

**Figure 1:**
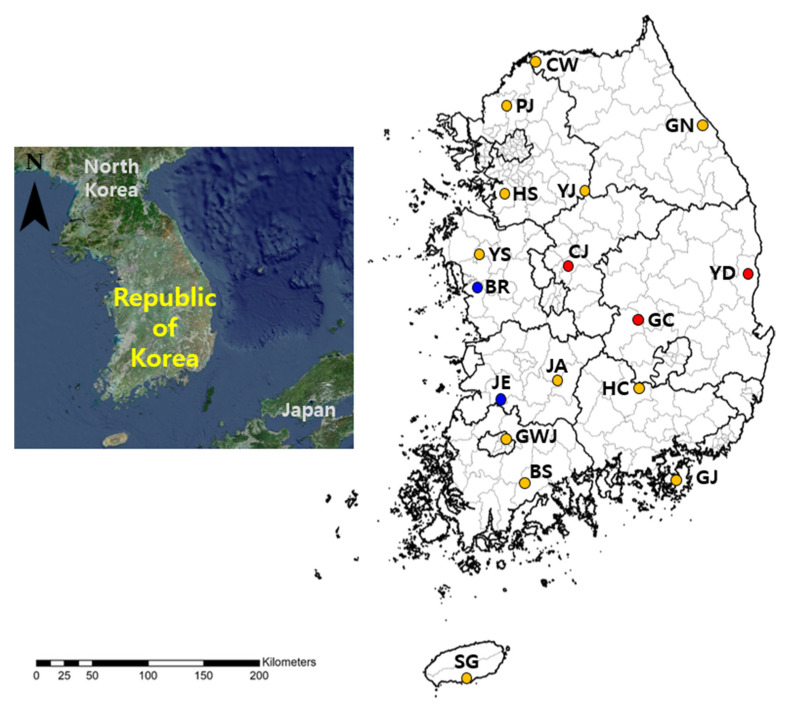
Geographic distribution of sampling sites for trombiculid mites collected from wild rodents in the Republic of Korea (ROK) in April 2025. Colored circles indicate the detection results for Orthohantaviruses in trombiculid mites using real-time polymerase chain reaction (PCR) and reverse transcription (RT)-nested PCR; red circles indicate the detection of *Orthohantavirus hantanense* by real-time PCR and RT-nested PCR; blue circles, detection of *O. jejuense*; yellow circles, Orthohantavirus-negative sites. CW, Cheorwon; PJ, Paju; GN, Gangneung; HS, Hwaseong; YJ, Yeoju; YS, Yesan; CJ, Cheongju; BR, Boryeong; YD, Yeongdeok; GC, Gimcheon; JA, Jinan; JE, Jeongeup; HC, Hapcheon; GWJ, Gwangju; BS, Boseong; GJ, Geoje; SG, Seogwipo.

Trombiculid mites were collected from wild rodents captured at 17 sites nationwide in April 2025 (Figure 1). Each collection site included five different environmental habitats, including rice fields, crop fields, reservoirs, waterways, and hillsides. Sherman folding live traps (3 × 3.5 × 9 inches, BioQuip, Gardena, CA, USA), baited with peanut butter-spread biscuits, were installed at 20 points at 5–10 m intervals in each environmental habitat. The traps were collected the following morning and transported to the laboratory. Captured rodents were euthanized using compressed carbon dioxide (CO_2_) and identified using taxonomic keys [19]. Lung and kidney tissues were collected in separate sterile tubes and stored at −70 °C until analysis. To collect ectoparasites, euthanized rodents were suspended for 24 h over a Petri dish filled with tap water. The fallen trombiculid mites were recovered from the water surface using a fine brush, and only living mites were selectively maintained in a mite-rearing container with charcoal and plaster mixture [20] to obtain fresh RNA for extraction. Approximately one-third (33%) of the collected trombiculid mites were randomly selected for molecular analysis. Other ectoparasites, such as ticks, were also collected, identified using taxonomic keys [21], and stored in 70% ethanol at −20 °C for subsequent molecular analysis.

### 2.3. RNA Extraction and Polymerase Chain Reaction (PCR) Amplification

Randomly selected trombiculid mites were pooled (up to 10 individuals per pool) according to their host and collection sites. Samples were placed in Precellys 2 mL Hard Tissue Reinforced Ceramic Beads kit (CK28-R) containing a lysis buffer and homogenized using a Precellys Evolution homogenizer (Bertin Technologies, Montigny-le-Bretonneux, France). The homogenates were centrifuged at 25,000× *g* for 10 min, and 100 μL of the supernatant was used for RNA extraction using a MagMAX *mir*Vana Total RNA Isolation kit (Applied Biosystems, Foster City, CA, USA) with a KingFisher Flex system (Thermo Fisher, Waltham, MA, USA) according to the manufacturer’s instructions. To screen the Orthohantaviruses, quantitative real-time PCR (qPCR) was conducted using a PowerChek^™^ HTNV/SEOV Real-time PCR Kit (KOGENE Biotech, Seoul, Republic of Korea) on a QuantStudio 5 instrument (Thermo Fisher Scientific, Inc., Waltham, MA, USA). Positive RNA samples from the screening were used for cDNA synthesis with a RevertAid First Strand cDNA synthesis Kit (Thermo Fisher, Waltham, MA, USA) and an oligonucleotide primer, OSM55 (5′-TAGTAGTAGACTCC-3′), designed from the conserved terminal 3′ and 5′ of the S, M, and L segments of Orthohantaviruses [22]. Nested PCR was conducted using Orthohantavirus-specific primers targeting the L segment (external primer; HAN-L-F1, 5′-ATGTAYGTBAGTGCWGATGC-3′ and HAN-L-R1, 5′-AACCADTCWGTYCCRTCATC-3′ and internal primer; HAN-L-F2, 5′-TGCWGATGCHACIAARTGGTC-3′ and HAN-L-R2, 5′-GCRTCRTCWGARTGRTGDGCAA-3′) [8]. The first PCR was performed by adding 5.0 μL of cDNA to AccuPower^®^ HotStart PCR PreMix (Bioneer, Daejeon, Republic of Korea) and incubating at 95 °C for 5 min, followed by 35 cycles of 95 °C for 30 s, 49 °C for 30 s, and 72 °C for 45 s, with a final amplification at 72 °C for 5 min. The secondary nested PCR used 5 μL of the primary PCR products under the following conditions: 95 °C for 5 min, followed by 30 cycles of 95 °C for 20 s, 54 °C for 20 s, 72 °C for 30 s, and a final extension step at 72 °C for 5 min. As an experimental positive control, *O. hantanense* NCCP 43298 was provided by the National Collection of Pathogens of Korea, and its cDNA was included in each PCR set with the negative control. Amplified PCR products of the expected size (391 bp) were confirmed using a QIAxcel Advanced System with the QIAxcel DNA screening kit (2400) (QIAGEN GmbH, Hilden, Germany). The primary screening for Orthohantavirus was performed on trombiculid mite pools. For any Orthohantavirus-positive pool, a secondary analysis was then conducted individually on the host rodent’s tissues (lung and kidney) and ectoparasites.

### 2.4. Molecular Identification of Rodents and Ticks

To confirm the species of host rodents and collected ticks, molecular identification was performed by amplifying the cytochrome b (*cytB*) [23] and cytochrome c oxidase I (*COI*) [24] genes. Total RNA was used as the template for a SuperScript™ III One-Step RT-PCR with Platinum™ *Taq* (Thermo Fisher Scientific, Inc., Waltham, MA, USA). Each RT-PCR mixture contained 1 μL of each oligonucleotide primer (10 pmol/μL) and 5 μL of the genomic RNA template. Reactions conditions were an initial step of 30 min at 55 °C and 2 min at 94 °C, followed by 35 cycles of 15 s at 94 °C, 30 s at 48 °C (*cytB*) or 60 s at 40 °C (*COI*), and 60 s at 72 °C, with final extension of 5 min at 72 °C yielding a predicted size of 1140 bp and 710 bp, respectively.

### 2.5. Sequencing and Phylogenetic Analysis

**Figure 2:**
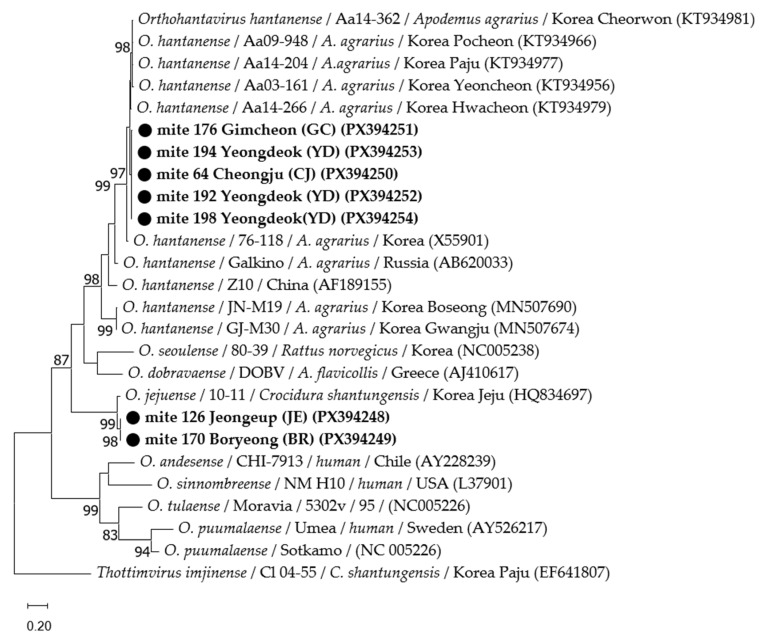
Phylogenetic analysis of *Orthohantavirus* species based on the partial L segment (352 bp). The analysis includes sequences from GenBank and Orthohantavirus-positive trombiculid mite pools collected from wild rodents in the ROK. The sequences identified in this study are indicated with black circles (●). The phylogenetic tree was constructed using the maximum likelihood method based on the Tamura 3-parameter model. Rate variation among sites was modeled using a gamma distribution with invariant sites (G + I). Numbers on the nodes indicate bootstrap support values based on 1000 replications. Bootstrap values < 80% are not shown.

All positive PCR products were sequenced in both directions using each PCR primer set by a commercial sequencing service, SolGent (Daejeon, Republic of Korea). Consensus sequences were generated using Chromas software (v.2.33). Sequences were compared with each Orthohantavirus, rodent, and tick species sequences reported in GenBank (National Center for Biotechnology Information, NCBI, Bethesda, MD, USA) and aligned using MEGA11 (v.11.0.13) with the MUSCLE (multiple sequence comparison by log-expectation) algorithm. Phylogenetic trees were constructed using the maximum likelihood method based on the best-fit nucleotide substitution models: the Tamura 3-parameter model for the Orthohantavirus L segment and the general time-reversible model for the rodent *cytB* and tick *COI* genes. Bootstrap analysis was replicated 1000 times to improve the confidence level of the phylogenetic tree. The GenBank accession numbers of the sequences obtained in this study are presented in Figure 2.

## 3. Results

### 3.1. Trombiculid Mite Collection and Orthohantavirus Minimum Infection Rate (MIR)

A total of 128 wild rodents were captured across 17 collection sites (Figure 1). The captured species were predominantly *Apodemus agrarius* (striped field mouse; 92.2%, *n* = 118), followed by *Crocidura* spp. (white-toothed shrew; 7.0%, *n* = 9), and *Craseomys regulus* (royal vole; 0.8%, *n* = 1). Of the 4963 trombiculid mites detached from these rodents, 1660 were randomly selected and grouped into 204 pools for Orthohantavirus screening (Table 1). OrthoHantavirus RNA was detected in seven pools, resulting in an overall MIR of 0.4 (7 positive pools/1660 tested mites). Among the collection sites, the highest MIR was observed in Boryeong (7.7, 1 pool/13 mites), followed by Yeongdeok (2.7, 3 pools/113 mites), Jeongup (1.1, 1 pool/94 mites), Gimcheon (0.7, 1 pool/147 mites), and Cheongju (0.4, 1 pool/229 mites). OrthoHantavirus species identification revealed that *O. hantanense* was found in Cheongju, Gimcheon, and Yeongdeok, whereas *O. jejuense* was identified in the Boryeong and Jeongeup regions (Table 1).

### 3.2. Orthohantavirus Detection in Host Rodents and Other Ectoparasites

The seven Orthohantavirus-positive trombiculid mite pools originated from seven different host rodents, six from *A. agrarius* and one from *C. regulus* (Table 2). Of these seven host rodent specimens, four *A. agrarius* specimens (one from Boryeong and three from Yeongdeok) tested positive for Orthohantavirus RNA during qPCR screening but no amplicons were detected by the subsequent RT-nested PCR, suggesting a low viral load. Notably, the host rodents with the Orthohantavirus-positive mite pools from Cheongju, Jeongeup, and Gimcheon were negative on qPCR. Other ectoparasites collected from these seven host rodents, including five *Ixodes nipponensis* nymphs, one mesostigmatid mite, one flea, and one louse, were negative for Orthohantaviruses. These results suggest host-independent Orthohantavirus infections in the trombiculid mites from Cheongju, Jeongeup, and Gimcheon.

### 3.3. Phylogenetic Analyses

A phylogenetic analysis was constructed based on partial L segment sequences obtained from the seven positive trombiculid mite pools and reference sequences from GenBank (Figure 2). The five sequences identified as *O. hantanense* from Cheongju, Gimcheon, and Yeongdeok shared 99.1–100% identity and formed clusters with other *O. hantanense* previously isolated in the ROK. The two sequences identified as *O. jejuense* from Boryeong and Jeongeup showed 94.8–99.7% similarity to *O. jejuense* 10-11 (HQ834697) isolated from Jeju in the ROK. Additionally, the species sequences from the seven host rodents and five *Ixodes nipponensis* obtained in this study clustered closely with their respective reference sequences (Supplementary Figure S1).

## 4. Discussion

This study is significant as it provides the first molecular detection of *Orthohantavirus hantanense* and *O. jejuense* in trombiculid mites detached from wild rodents in the ROK. The MIR for Orthohantaviruses in trombiculid mites was 0.4 per 100 mites (7 positive pools/1660 mites), which is a notable MIR compared to other major vector-borne diseases in previous studies. For instance, a study reported an MIR of 0.09 per 100 mites for the causative agent of scrub typhus in the trombiculid mite population [15]; another study reported 0.2 per 100 ticks for severe fever with thrombocytopenia syndrome (SFTS) virus in the tick population [25]. Considering that trombiculid mites and ticks are the primary vectors and reservoirs for scrub typhus [20] and SFTS [26], respectively, MIR of Orthohantavirus in trombiculid mites found in this study can be considered relatively high. Although the MIR can be influenced by various factors such as the survey environment, timing, and the number of specimens tested, and tends to be an underestimate of individual infection rates [27], this result clearly indicates that Orthohantavirus is considerably present within trombiculid mite populations in the ROK.

In this study, Orthohantavirus was detected in four of the seven host rodents with Orthohantavirus-positive trombiculid mite pools. This suggests the possibility of viral transmission between the trombiculid mites and their host rodents. Particularly, the pathway of virus acquisition from the host to the trombiculid mite can be inferred from previous studies. Unlike hematophagous ticks, trombiculid mites are known to primarily ingest the host’s decomposed tissue and lymph fluid [16]. However, several studies have confirmed through occult blood tests [28] and pathological observations of skin tissue [29] that trombiculid mites can ingest small amounts of blood, which indicates that a pathway exists for acquiring the virus from an infected host.

There are several points to consider in this study. First, the host rodent specimens in which Orthohantavirus was detected by real-time PCR were not amplified by RT-nested PCR; this could be due to a low viral load, differences in the sensitivity of the experimental methods, or the influence of PCR inhibitors, requiring further analysis. Second, Orthohantavirus was not detected in other ectoparasites, including ticks, a flea, and a louse, collected from these rodents. However, this result may be due to RNA degradation of ectoparasites, excluding trombiculid mites, during storage in 70% ethanol.

The most critical finding of this study was the detection of Orthohantavirus in trombiculid mites from Cheongju, Jeongeup, and Gimcheon, where the host rodents tested negative for the Orthohantavirus, demonstrating that trombiculid mites can be infected with Orthohantavirus independently of their hosts. Such host-independent infection implies the existence of a new transmission cycle for Orthohantavirus by trombiculid mites, separate from the traditionally known route. Previous studies support the possibility of such a transmission cycle. In China, Orthohantaviruses identical to wild rodents and patients were also detected in trombiculid mites [17]. Additionally, Orthohantaviruses were also found in free-living trombiculid mites in the soil, not parasitic on rodents in the United States [18]. This key finding of host-independent infection strongly suggests that trombiculid mites could act as independent reservoirs for Orthohantavirus through transovarial transmission, considering their life cycle, where the larva parasitizes a host only once.

This study had several limitations. The infectivity and viability of the detected viral RNA were not confirmed. Additionally, the vectorial capacity of the trombiculid mites and the occurrence of transovarial transmission were not directly proven. Therefore, future research should focus on investigating Orthohantavirus infection in free-living soil trombiculid mites to confirm transovarial transmission and on elucidating the mechanisms of pathogen acquisition and transmission, including hematophagy, to clarify the precise role of trombiculid mites in the Orthohantavirus transmission cycle.

## 5. Conclusions

This nationwide survey provides the first molecular evidence of *O. hantanense* and *O. jejuense* infection in trombiculid mites in the ROK. The discovery of Orthohantavirus in trombiculid mites from uninfected rodents is a crucial finding, raising the possibility that these trombiculid mites may act as independent reservoirs, potentially through transovarial transmission, and emphasizing the need for further research into their vectorial capacity.

## Figures and Tables

**Table 1:** Number of collected trombiculid mites from wild rodents in the Republic of Korea by region (April 2025).

Region	No. of Collected Rodents	No. of Collected Mites	No. of Tested Mites	No. of Tested Pools	No. of Positive Pools	MIR (%)
Cheorwon	10	605	230	27	0	0
Paju	2	246	91	10	0	0
Gangneung	10	186	65	12	0	0
Hwaseong	2	242	119	13	0	0
Yeoju	6	718	115	14	0	0
Yesan	3	141	61	7	0	0
Cheongju	13	781	229	29	1	0.4
Boryeong	6	26	13	3	1	7.7
Yeongdeok	8	349	113	14	3	2.7
Gimcheon	5	366	147	16	1	0.7
Jinan	8	126	40	6	0	0
Jeongeup	5	281	94	11	1	1.1
Hapcheon	6	291	125	14	0	0
Gwangju	5	120	23	4	0	0
Boseong	5	61	9	2	0	0
Geoje	5	374	169	18	0	0
Seogwipo	29	50	17	4	0	0
Total	128	4963	1660	204	7	0.4

MIR (%), minimum infection rate (number of positive pools/total number of tested mites × 100).

**Table 2:** Number of rodents, trombiculid mites, and ectoparasites tested for Orthohantavirus.

Species	No. of Collected Rodents	No. of Rodents with HP Mite Pools	No. of HP Rodents with HP Mite Pools	No. of Ectoparasites from Rodents with HP Mite Pools				
				*Ixodes* *nipponensis*	*Mesostigmata* spp.	*Pulicidae* spp.	*Anoplura* spp.	Total
*Apodemus agrarius*	118	6	4	5	1	1	1	8
*Craseomys* *regulus*	1	1	0	0	0	0	0	0
*Crocidura* spp.	9	0	0	0	0	0	0	0

HP, Orthohantavirus-Positive.

## Data Availability

The datasets analyzed during the current study are available from the corresponding author on reasonable request.

## Supplementary Materials

The following supporting information can be downloaded at: https://www.mdpi.com/article/10.3390/pathogens14121260/s1, Figure S1: Phylogenetic analysis of host animals and ectoparasites.

## Abbreviations

The following abbreviations are used in this manuscript:

HPS	hantavirus pulmonary syndrome
SFTS	severe fever with thrombocytopenia syndrome
qPCR	quantitative polymerase chain reaction
ROK	Republic of Korea
HFRS	hemorrhagic fever with renal syndrome
MIR	minimum infection rate
