# Supplementary material for: First Molecular Detection of Orthohantaviruses (Orthohantavirus hantanense and O. jejuense) in Trombiculid Mites from Wild Rodents in the Republic of Korea
Source: Pathogens. 2025 Dec 9;14(12):1260. doi: 10.3390/pathogens14121260 (PMC12735936; doi:10.3390/pathogens14121260)
Supplement: Supplementary file 1 [file pathogens-14-01260-s001.zip › pathogens-4002894-supplementary.pdf]

Supplementary Materials

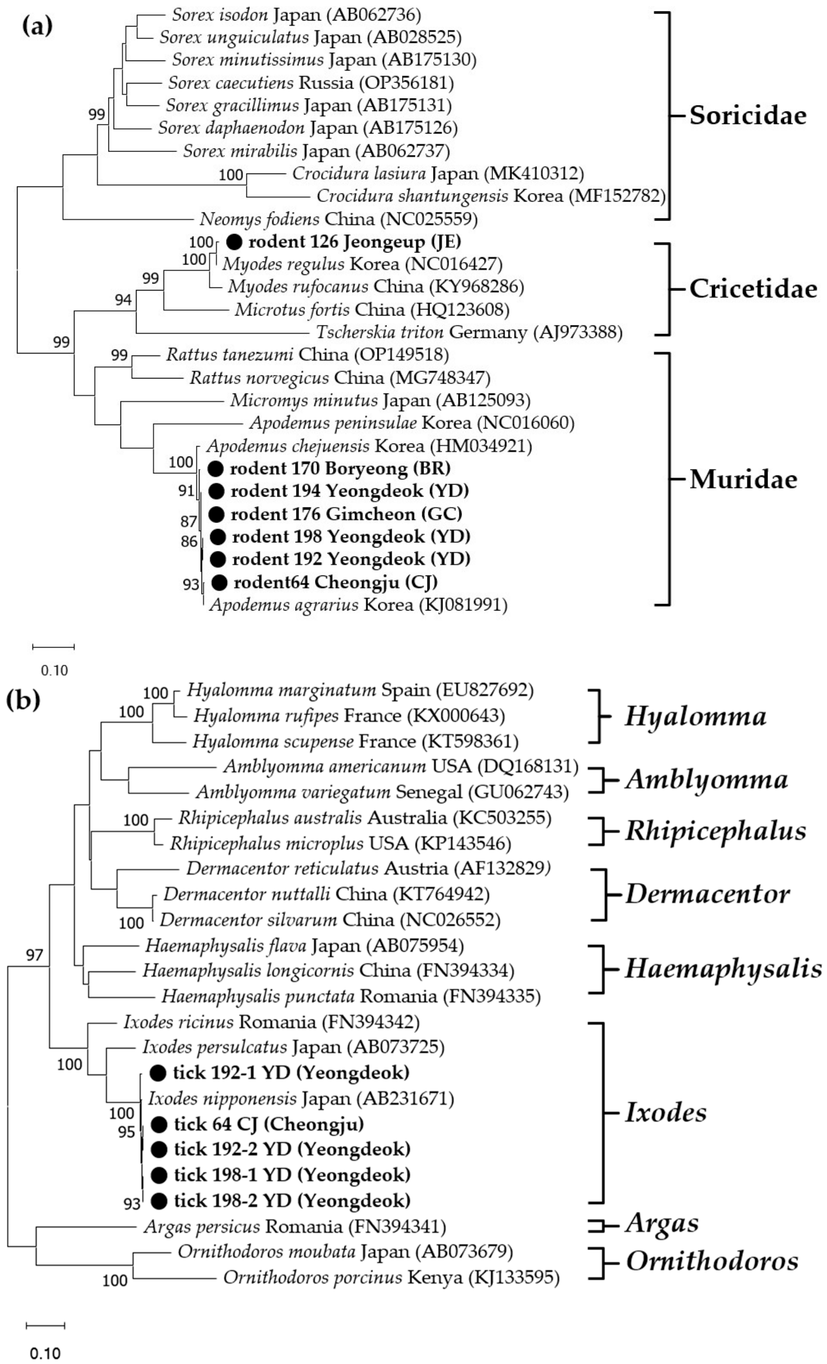

Figure S1. Phylogenetic analysis of host animals and ectoparasites. (**a**) Phylogenetic tree for host rodents based on the partial cytochrome b (*cytB*) gene (726 bp) and (**b**) ticks based on cytochrome c oxidase subunit I (*COI*) genes (596 bp). Sequences identified in this study are indicated with black circles (●). The phylogenetic trees were constructed using the maximum likelihood method based on the general time-reversible model. Rate variation among sites was modeled using a gamma distribution with invariant sites (G + I). The numbers on the nodes indicate bootstrap support values based on 1,000 replications. Bootstrap values <80% are not shown.
